# Spike protein of SARS‐CoV‐2 Omicron (B.1.1.529) variant has a reduced ability to induce the immune response

**DOI:** 10.1038/s41392-022-00980-6

**Published:** 2022-04-09

**Authors:** Cai He, Xuemei He, Jingyun Yang, Hong Lei, Weiqi Hong, Xiangrong Song, Li Yang, Jiong Li, Wei Wang, Guobo Shen, Guangwen Lu, Xiawei Wei

**Affiliations:** Laboratory of Aging Research and Cancer Drug Target, State Key Laboratory of Biotherapy and Cancer Center, National Clinical Research Center for Geriatrics, West China Hospital, Sichuan University, 610041 Chengdu, China

**Keywords:** Infectious diseases, Immunotherapy

**Dear Editor**,

The severe acute respiratory syndrome coronavirus 2 (SARS-CoV-2) variant Omicron (B.1.1.529) has attracted great concerns since its identification in South Africa. Omicron is the fifth variant of concern (VOC) after Alpha (B.1.1.7), Beta (B.1.351), Gamma (P.1) and Delta (B.1.617.2), and set a record with the shortest duration from variants of interest (VOI) to VOC so far. Within 2 months after its first report, over 80% of global sequenced samples are verified as Omicron according to GISAID (https://cov-spectrum.org/explore/World/AllSamples/from=2021-12-15&to=2022-01-15/variants?pangoLineage=B.1.1.529*), indicating the dominant prevalence of Omicron and faster transmissibility than Delta variant. Omicron variant harbors >30 substitutions/deletions/insertions in the spike, including 15 substitutions in receptor-binding domain (RBD) according to GISAID (https://covariants.org/variants/21K.Omicron). The decreased neutralizing ability of therapeutic antibodies, sera from convalescents and vaccine recipients against Omicron variant and impaired vaccine effectiveness have prompted the selection of Omicron spike as the antigens of vaccine development.^1^ Therefore, high expectations have been pinned on the protective effects of vaccines based on mutant Omicron spike.

Spike has been prioritized for vaccine development because of its essential functions in host receptor binding and unique feature of relatively conserved characteristics, good immunogenicity for neutralizing antibody induction, as well as effective target for T-cell responses.^2^ Spike is composed of S1 and S2 subunits, and RBD-included S1 is largely responsible for the immunogenicity of spike. Therefore, we constructed recombinant protein vaccines composed of MF59 adjuvant and wild-type spike subunit 1 protein (S1-WT) or Omicron S1 protein (S1-Omicron). NIH mice were immunized with S1-WT and S1-Omicron recombinant protein vaccine candidates on day 0, 14, 28, respectively. Sera samples were collected 14 days after the last administration. The antibody response assay results showed that the antibody titers of S1-WT- and RBD-WT-specific IgG antibodies (GMT: 1.1 × 10^7^ and 5.0 × 10^6^, respectively) in S1-WT protein immunized mice were much higher than that of S1-Omicron- and RBD-Omicron-specific IgG antibodies (GMT: 6.6 × 10^6^ and 1.4 × 10^6^, respectively) in S1-Omicron immunized mice. Both the S1-WT and S1-Omicron immunized sera showed reduced cross-reaction with S1-WT or S1-Omicron antigens (Fig. 1a and Supplementary Fig. S1a, b). These results revealed that recombinant S1-WT protein vaccine could induce stronger antibody response against wild-type S1 and RBD than the response induced by S1-Omicron protein vaccination against S1-Omicron and RBD-Omicron, implying the profoundly lower humoral immunity induced by S1-Omicron protein than S1-WT protein.

**Fig. 1:**
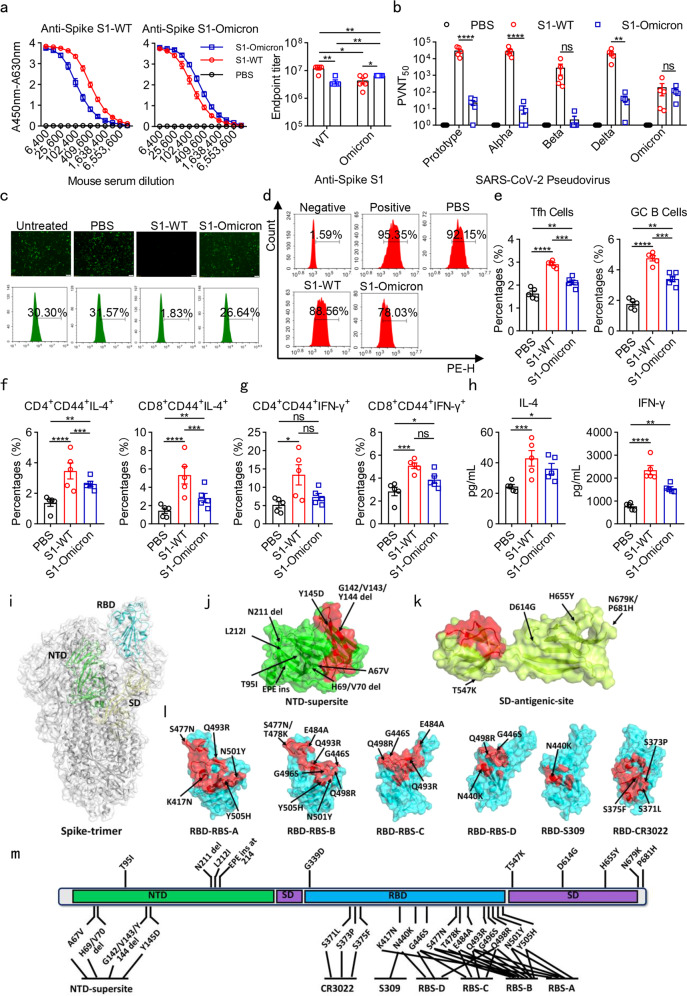
Recombinant Omicron S1 protein vaccine induced weak humoral and cellular immunity in mice. **a** Estimation of the binding ability of sera from immunized with PBS, S1-WT, and S1-Omicron proteins to S1-WT (left) and S1-Omicron (middle)-coated antigens by ELISA. The absorbance was read at 450–630 nm. Antibody titers of S1-WT or S1-Omicron IgG (Right) in sera collected from NIH mice immunized with S1-WT or S1-Omicron vaccine. **b** PVNT_50_ of the immune sera against prototype, Alpha, Beta, Delta, and Omicron pseudoviruses. The value was defined as the inverse dilution that achieved 50% neutralization and calculated by GraphPad Prism 8.0. **c** The blocking effects of sera from immunized mice on the infection of SARS-CoV-2 EGFP/Luciferase-expressing pseudovirus into 293T/ACE2 cells. Immune sera samples were diluted at 1:90. **d** Representative flow cytometry showing the blockade of RBD-Omicron binding to ACE2 by immune sera. The dilution ratio of sera was 1:90. Negative: without RBD-Omicron protein; Positive: without sera; PBS: sera from mice immunized with PBS; S1-WT: sera from mice immunized with S1-WT protein; S1-Omicron: sera from mice immunized with S1-Omicron protein. **e** Flow cytometric analyses of Tfh and GC B cells in spleens. Mice were sacrificed 14 days after the last immunization with PBS, S1-WT, and S1-Omicron recombinant protein vaccines. The spleen lymphocytes were isolated with lymphocyte isolation solution by density gradient centrifugation and stained with cell-surface markers. Tfh cells were identified as CD3^+^CD19^−^CD4^+^CXCR5^+^PD-1^+^. GC B cells were CD3^−^CD19^+^CD95^+^GL7^+^. **f**, **g** T-cell recall responses in spleen lymphocytes separated from S1-WT or S1-Omicron immunized mice. NIH mice immunized with PBS, candidate S1-WT or S1-Omicron vaccines were euthanized 14 days after the last immunization. Spleen lymphocytes were isolated and stimulated with recombinant S1-WT or S1-Omicron proteins for 72 h. S1-WT- or S1-Omicron-reactive memory CD4 or CD8 T cells were measured by gating on CD45R^−^MHC-II^−^CD44^+^, as well as CD4^+^ or CD8^+^. **h** Cytokines IFN-γ and IL-4 produced by isolated spleen lymphocytes in culture supernatant were estimated by ELISA after stimulation with 10 μg/ml recombinant S1-WT or S1-Omicron proteins for 72 h. **i** An overview of the SARS-CoV-2 spike-trimer structure. The N-terminal domain (NTD), receptor-binding domain (RBD), and subdomain (SD-1 and -2) in one protomer are colored green, cyan, and pale yellow, respectively, and marked. **j**–**l** A surface view of the known antigenic-sites/epitopes in spike. In each case, one representative neutralizing antibody targeting the site is selected to depict the epitopes (highlighted in red). The mutations identified in the Omicron variant (based on covariant 21 K at address http://covariants.org) are labeled and further marked with arrows. **j** The supersite in NTD (based on antibody S2M28). **k** An antigenic site identified in SD (based on S3H3). **l** The antigenic-sites RBS-A (based on CB6), RBS-B (based on CV07-250), RBS-C (based on CV07-270), RBS-D (based on REGN10987), S309 (based on C135), and CR3022 (based on H014) in RBD. **m** A schematic view summarizing the Omicron spike-mutations that are in the close proximity to or are directly located within the above-listed antigenic-sites/epitopes. Data are mean ± S.E.M. *p*-values were determined by unpaired Student’s *t*-tests (*n* = 5 in each group). *p < 0.05, **p < 0.01, ***p < 0.001, ****p < 0.0001. *ns*: not significant

We further analyzed the 50% pseudovirus neutralization titers (PVNT_50_) of sera from S1-WT and S1-Omicron proteins immunized mice against prototype, Alpha, Beta, Delta and Omicron pseudoviruses. The results showed that S1-WT protein-induced significantly higher neutralization against prototype, Alpha, Delta pseudoviruses than S1-Omicron protein. The GMT in S1-WT immunized sera against prototype pseudovirus reached 2.6 × 10^4^, but reduced by 452 times when against Omicron pseudovirus (GMT: 58). Unexpectedly, the neutralization potency of the S1-Omicron-immunized sera was inefficient against the Omicron pseudovirus (GMT: 85). Moreover, sera from S1-Omicron immunized mice also displayed poor neutralization ability against other detected pseudoviruses (Fig. 1b). The above results demonstrated that immunization with S1-Omicron protein could not elicit enough neutralizing antibodies to combat Omicron-included SARS-CoV-2 variants. Furthermore, we used EGFP/Luciferase-expressing prototype pseudovirus to identify the neutralizing ability of the immune sera. As shown in Fig. 1c, the numbers of EGFP-expressing cells were significantly decreased after the incubation of pseudovirus with the sera from S1-WT group while compared to the phosphate-buffered saline (PBS) control, indicating the excellent neutralization of the immune sera from S1-WT protein group against prototype pseudovirus. However, the sera from S1-Omicron group lost the neutralization ability against prototype pseudovirus. This result was also supported by the flow cytometry analysis (Fig. 1c, below panel). Moreover, after the vaccination of RBD-WT and RBD-Omicron proteins, the sera were collected and used to study the inhibitory efficiency of RBD binding to receptor ACE2. The results revealed that the immune sera from S1-WT and S1-Omicron vaccinated group could hardly block the binding of RBD-Omicron with 293T/ACE2 cells, which showed no significant difference compared to PBS group (Fig. 1d). However, sera from S1-WT protein immunized mice could specifically obstruct the binding of RBD-WT with 293T/ACE2 cells (Fig. S1c, d). The above results suggest that S1-Omicron protein could not elicit ideal neutralizing antibodies against these detected pseudoviruses, including Omicron.

Germinal center (GC) B cells can differentiate into antibody-secreting long-lived plasma cells and memory B cells, which is regulated by T follicular helper (Tfh) cells.^3^ Therefore, to elaborate the suboptimal neutralizing antibody levels induced by S1-Omicron vaccine candidate, we isolated spleen lymphocytes from PBS, S1-WT, and S1-Omicron protein immunized mice to determine the Tfh and GC B cells. The flow cytometry results showed that the percentages of Tfh and GC B cells in S1-WT group were remarkably elevated compared to PBS and S1-Omicron group, which might be associated with the reduced neutralizing antibody production in S1-Omicron immunized mice (Fig. 1e). We further re-stimulated the isolated spleen lymphocytes with 10 μg/ml recombinant S1-WT or S1-Omicron proteins for 72 h to examine T cells responses. The results showed that S1-WT protein stimulation recalled more antigen-specific reactive memory CD4^+^ or CD8^+^ T cells with IL-4 production compared to PBS and S1-Omicron group, regardless of significantly higher T-cell response observed in S1-Omicron group while compared with that of PBS group (Fig. 1f). The percentages of IFN-γ-secreting memory CD4^+^ or CD8^+^ T cells in S1-WT group were increased after the treatment of S1-WT protein, while only IFN-γ-secreting memory CD8^+^ T cells were increased in S1-Omicron group compared to that of the PBS control (Fig. 1g). Correspondingly, the levels of IFN-γ and IL-4 in the supernatant of spleen lymphocytes after incubation with recombinant S1-WT or S1-Omicron for 72 h significantly elevated in S1-Omicron group while compared to PBS control, and which were further increased in S1-WT groups (Fig. 1h). These results indicated that both S1-WT or S1-Omicron protein vaccine candidates could generate Th1- and Th2-mediated immune responses, but S1-Omicron was less likely to trigger immune response in mice compared to S1-WT protein.

Several studies have shown that the prevalent Omicron mutations could dramatically change the antigenic features of the viral spike, leading to significantly reduced neutralization. Within the spike trimer, the N-terminal domain (NTD), RBD and subdomains 1 and 2 (SD1 and SD2) in S1 are both solvent accessible and can therefore be targeted by neutralizing antibodies (Fig. 1i). In NTD, multiple neutralizing antibodies have been shown to recognize a relatively concentrated region, leading to an antigenic supersite. The Omicron spike-substitutions (e.g., A67V, Y145D) and –deletions (e.g., H69/V70 del, G142/V143/Y144 del) in NTD are either in the close proximity to or directly located within the supersite (Fig. 1j, m), resulting in altered antigenicity and diminished/abolished antibody binding. For RBD, it has been featured with several hotspot antigenic-sites (e.g., RBS-A, -B, -C, -D, S309, and CR3022), to which a majority of the currently characterized RBD-specific neutralizing antibodies might bind. It is notable that up to 15 Omicron spike-substitutions have occurred in the RBD region. These mutations are evidently each located to at least one of the antigenic-sites (e.g., S371L, S373P, S375F) and in most cases to two or more sites (e.g., N440K, G446S, S477N, T478K, E484A, Q493R, Q498R, N501Y, Y505H) (Fig. 1l, m). While RBD is the predominant target of neutralization, such residue-substitutions and the subsequent changes to the antigenic profile of RBD would lead to significantly decreased recognition and binding by neutralizing antibodies. Unlike those targeting NTD or RBD, neutralizing antibodies that bind to SD currently are not well characterized. One of these (S3H3), which recognizes SD1, seems not to be affected by the Omicron mutations (Fig. 1k). Nevertheless, the antigenicity of SD is likely to be limited, thus only a few SD-specific neutralizing antibodies have now been successfully identified. Taken together, the marked antigenic changes in Omicron spike, especially those in NTD and RBD, would notably cause diminished neutralization, which might be partly responsible for the reduced cross-reactivity of S1-WT and S1-Omicron proteins immunized sera against prototype and Omicron pseudoviruses. It is also noteworthy that a recent study has shown that Omicron RBD is of decreased thermostability and at the same time is more susceptible to protease-digestion.^4^ While RBD is the key immunogen for vaccine-induced protection, the altered antigenicity (as illustrated by the wide distribution of the Omicron mutations in known antigenic-sites/epitopes) and the changed protein characteristics (e.g., less thermostable) might explain the reduced response of the Omicron S1 antigen during immunization.

In summary, our data elucidate that recombinant protein vaccine based on Omicron S1 elicited an impaired serologic response and exerted drastically reduced neutralizing activity against SARS-CoV-2 wild-type or Omicron-included variants, while the T-cell response remained to be initiated, but significantly weaker than the wild-type S1 protein-induced ones. This inefficient humoral immunity of Omicron S1 protein vaccine might elaborate, at least partly, the recent clinical findings that antibody titers in Omicron breakthrough individuals were substantially lower than those after Delta infection.^5^ Strikingly, Gagne et al.^6^ discovered that 2 weeks after boost with mRNA-1273 or mRNA-Omicron, the neutralizing antibody titers against Omicron were 2980 and 1930. They pointed out that vaccination with Omicron boost might not increase antibody titers compared to the wild-type mRNA-1237 boost. Therefore, Omicron S1 protein might not be a prioritized antigen for designing and developing vaccines against Omicron variant. Some optimization strategies might be carefully considered when designing and developing protein vaccines against Omicron variant in the future. The principal one is the length and structure of candidate antigens. Longer spike, including full length of spike or sub-full-length extracellular domain, and spike with similar physiological structure (e.g., spike trimer or virus-like particle) might improve the immunogenicity of antigens. In addition, spike could be used to form fusion proteins with other proteins or peptides with adjuvant effects, such as tetanus toxin, to form a new antigen with better immunogenicity, so as to increase the protective effects of vaccines. Naturally, the selection of a proper adjuvant for the formation of recombinant vaccine is also essential for the improvement of vaccine efficiency. Moreover, the combination of recombinant protein subunit vaccines with other types of COVID-19 vaccines or sequential vaccination of different kinds of COVID-19 vaccines might also be optimization strategies to prevent and overcome the Omicron.

## Supplementary information

Supplementary Materials for Spike protein of SARS‐CoV‐2 Omicron (B.1.1.529) variant have a reduced ability to induce the immune response

Supplementary Materials for Spike protein of SARS‐CoV‐2 Omicron (B.1.1.529) variant have a reduced ability to induce the immune response

## Data Availability

The data included in this study are available upon request from the corresponding author.
