# Supplementary material for: Spike protein of SARS‐CoV‐2 Omicron (B.1.1.529) variant has a reduced ability to induce the immune response
Source: Signal Transduct Target Ther. 2022 Apr 9;7:119. doi: 10.1038/s41392-022-00980-6 (PMC8994023; doi:10.1038/s41392-022-00980-6)
Supplement: Supplementary file 1 — Supplementary Materials for Spike protein of SARS‐CoV‐2 Omicron (B.1.1.529) variant have a reduced ability to induce the immune response [file 41392_2022_980_MOESM1_ESM.docx]

Supplementary Materials for

Spike protein of SARS‐CoV‐2 Omicron (B.1.1.529) variant have a reduced ability to induce the immune response

Cai He^1,+^, Xuemei He^1,+^, Jingyun Yang^1,+^, Hong Lei^1^, Weiqi Hong^1^, Xiangrong Song^1^, Li Yang^1^, Jiong Li^1^, Wei Wang^1^, Guobo Shen^1^, Guangwen Lu^1*^, Xiawei Wei^1*^

*1. Laboratory of Aging Research and Cancer Drug Target, State Key Laboratory of Biotherapy and Cancer Center, National Clinical Research Center for Geriatrics, West China Hospital, Sichuan University, Chengdu, 610041, China*

^+^ These authors contributed equally to this work

* Correspondence: Guangwen Lu ([lugw@scu.edu.cn](mailto:lugw@scu.edu.cn)), Xiawei Wei (xiaweiwei@scu.edu.cn)

**This PDF file includes:**

Materials and Methods

Supplemental Figure S1

**Materials and Methods**

1. **Information of recombinant proteins**

The S1 of SARS-CoV-2 wild-type spike (S1-WT) and S1 of Omicron spike (S1-Omicron) were provided by ACRO Biosystems. All S1 proteins were expressed by human 293 cells (HEK293) with His Tag ranging from 16 to 685 amino acid site of spike. The mutations in S1-Omicron were A67V, HV69-70del, T95I, G142D, VYY143-145del, N211del, L212I, ins214EPE, G339D, S371L, S373P, S375F, K417N, N440K, G446S, S477N, T478K, E484A, Q493R, G496S, Q498R, N501Y, Y505H, T547K, D614G, H655Y, N679K, P681H. The endotoxin in all recombinant proteins in this study was lower than 1.0 EU per protein examined by LAL method.

1. **Vaccine formulation and mice vaccination**

The recombinant protein vaccine candidates were constructed with 10 μg recombinant proteins S1-WT or S1-Omicron and MF59 by mixing as emulsifiable mixture. Specific pathogen-free (SPF) female NIH mice (6-8 weeks) were purchased from HFK bioscience company (China, Beijing) for immunization. Mice were intramuscularly immunized with PBS, S1-WT or S1-Omicron vaccine candidates on days 0, 14 and 28, separately. Blood samples were collected 14 days after the last immunization and sera were isolated by centrifugation at 6000 rpm for 10 min. Sera were stored at -20℃ before use.

1. **Cell culture**

HEK293T cells were purchased from the American Type Culture Collection (ATCC). HEK293T cells with stable expression of human ACE2 (293T/ACE2) were constructed by our laboratory as previously reported.^1^ Cells were cultured in complete Dulbecco's modified Eagle's medium (DMEM, Gibco, USA), supplemented with 10 % fetal bovine serum (FBS, PAN-Biotech, Germany), streptomycin (100 μg/ml) and penicillin (100 U) (Gibco, USA) at 37 °C with 5% CO_2_.

1. **Enzyme-linked immunosorbent assays**

Recombinant S1-WT, S1-Omicron, RBD-WT and RBD-Omicron proteins were used as antigens to coat flat-bottom 96-well high binding plates (NUNC-MaxiSorp, Thermo Fisher Scientific). After coating overnight at 4℃, the plates were washed with phosphate buffered saline containing 0.1% Tween-20 (PBST), and blocked with 1% BSA for 1 h at room temperature. Then, diluted anti-mouse horseradish peroxidase (HRP) antibodies (Southern Biotech, USA) were added into each well and incubated for 1 h. After reaction with 3,3',5,5'-tetramethyl biphenyl diamine (TMB) substrate for 10 min at room temperature, the reaction was stopped with 50 μl/well of 1.0 M H_2_SO_4_ solution, and the absorbance was measured by 450 nm on a microplate reader.

1. **Blockade of** **RBD binding to 293T/ACE2**

The binding of RBD binding to the receptor ACE2 on 293T/ACE2 cells was performed as described previously^1^. Briefly, 0.4 μg/ml of RBD-WT-Fc, RBD-Omicron-Fc proteins were incubated with PBS or diluted mouse sera for 30 min at 37℃ and then added to the 293T/ACE2 cells. After the incubation at room temperature for 30 min, cells were washed with PBS and stained with PE-labeled anti-human IgG Fc (BioLegend, USA) at 4℃ for 30 min. Then, cells were detected and analyzed by NovoCyte Flow Cytometer (ACEA Biosciences).

1. **Pseudovirus neutralization assay**

The prototype, Alpha, Beta, Delta and Omicron pseudoviruses with EGFP/Luciferase-expressing were purchased from Genomeditech (China, Shanghai). The pseudovirus neutralization was performed as described previously.^1^ Briefly, The prototype, Alpha, Beta, Delta or Omicron pseudoviruses were pre-incubated with serum serially diluted from 1:90 for 1 h at 37 °C. Afterward, added 293T/ACE2 cells to the 96 wells plates and cells were cultured for another 48 h to express the reporter gene. Finally, removed the cell supernatant, added 100 µl lysis reagent with luciferase substrate (Promega, USA) to determine the relative light unit (RLU) by a multi-mode microplate reader (PerkinElmer, USA). Pseudovirus 50% neutralizing titers were expressed as the highest dilution that caused 50% inhibitory relative to the average of the virus control wells and calculated by a non-linear regression model (inhibitor versus normalized response) in GraphPad 8.0 software.

1. **Spleen lymphocytes isolation and stimulation**

Mice were euthanized 14 days after full immunization with PBS, S1-WT or S1-Omicron vaccine candidates. Spleen lymphocytes were isolated with lymphocyte isolation solution by density gradient centrifugation at 800 g for 30 min. The spleen lymphocytes were washed with PBS and plated into 12-well plates with a density of 1×10^6^ cells per well. Then, lymphocytes were stimulated with 10 μg/ml recombinant S1-WT or S1-Omicron proteins for 72 h. The lymphocytes were collected for flow cytometry analysis and the culture supernatants were harvested for IFN-γ and IL-4 detection.

1. **Flow cytometry**

The isolated spleen lymphocytes from immunized mice were stained with cell-surface markers at 4℃ for 30 min. The antibodies PerCP/Cyanine5.5-conjugated anti-mouse CD3, FITC-conjugated anti-mouse CD4, PE-conjugated anti-mouse CD19, Brilliant Violet 421-conjugated anti-mouse PD-1, APC-conjugated anti-mouse CXCR5, Brilliant Violet 421-conjugated anti-mouse CD19, FITC-conjugated anti-mouse GL7 and PE-CF-594-conjugated anti-mouse CD95 were used for Tfh and GC B cells analysis.

For T cell response assay, the spleen lymphocytes were stimulated with recombinant S1-WT or S1-Omicron proteins and brefeldin A (BFA, BD Biosciences) were added to block intracellular cytokine secretion before the harvest of the lymphocytes. Then, lymphocytes were stained with PerCP/Cyanine5.5-conjugated anti-mouse CD45R, PE-Cy7-conjugated anti-mouse MHCII, APC-conjugated anti-mouse CD4, FITC-conjugated anti-mouse CD8, Brilliant Violet 510-conjugated anti-mouse CD44 antibodies. Later, lymphocytes were fixed and permeabilized and stained with PE-conjugated anti-mouse IFN-γ and Brilliant Violet 421 anti-mouse IL-4 antibodies at room temperature for 1 hour. Cells were analyzed by the NovoCyte Flow Cytometer (ACEA Biosciences) and data were analyzed with NovoExpress software.

1. **Statistical analyses**

Statistical analyses were performed using GraphPad 8.0 software. Data are shown as mean ± S.E.M. *P* values were determined by unpaired Student’s t-tests. *p* < 0.05 was considered significant.

**References**

1 Yang, J. *et al.* A vaccine targeting the RBD of the S protein of SARS-CoV-2 induces protective immunity. *Nature* **586**, 572-577, doi:10.1038/s41586-020-2599-8 (2020).

**Supplementary Figure**

**Fig. S1** The binding ability of immune sera to RBD-WT and RBD-Omicron coated antigens and the blockade of immune sera against RBD-WT binding to ACE2. **a** Evaluation of the binding ability of sera from immunized with PBS, S1-WT and S1-Omicron proteins to RBD-WT and RBD-Omicron coated antigens by ELISA. The absorbance was read at 450-630 nm. **b** Antibody titers of RBD-WT or RBD-Omicron IgG in immune sera. **c, d** The blockade of RBD-WT binding to 293T/ACE2 by immune sera was analyzed by flow cytometry (**c**) and inhibition rate was calculated (**d**). The immune sera samples were diluted at 1:90 before use. Negative: without RBD-Omicron protein; Positive: without sera; PBS: sera from mice immunized with PBS; S1-WT: sera from mice immunized with S1-WT protein; S1-Omicron: sera from mice immunized with S1-Omicron protein. Data are presented as mean ± S.E.M. *P* values were determined by unpaired Student’s t-tests (n=5 in each group). **P < 0.05*.

**
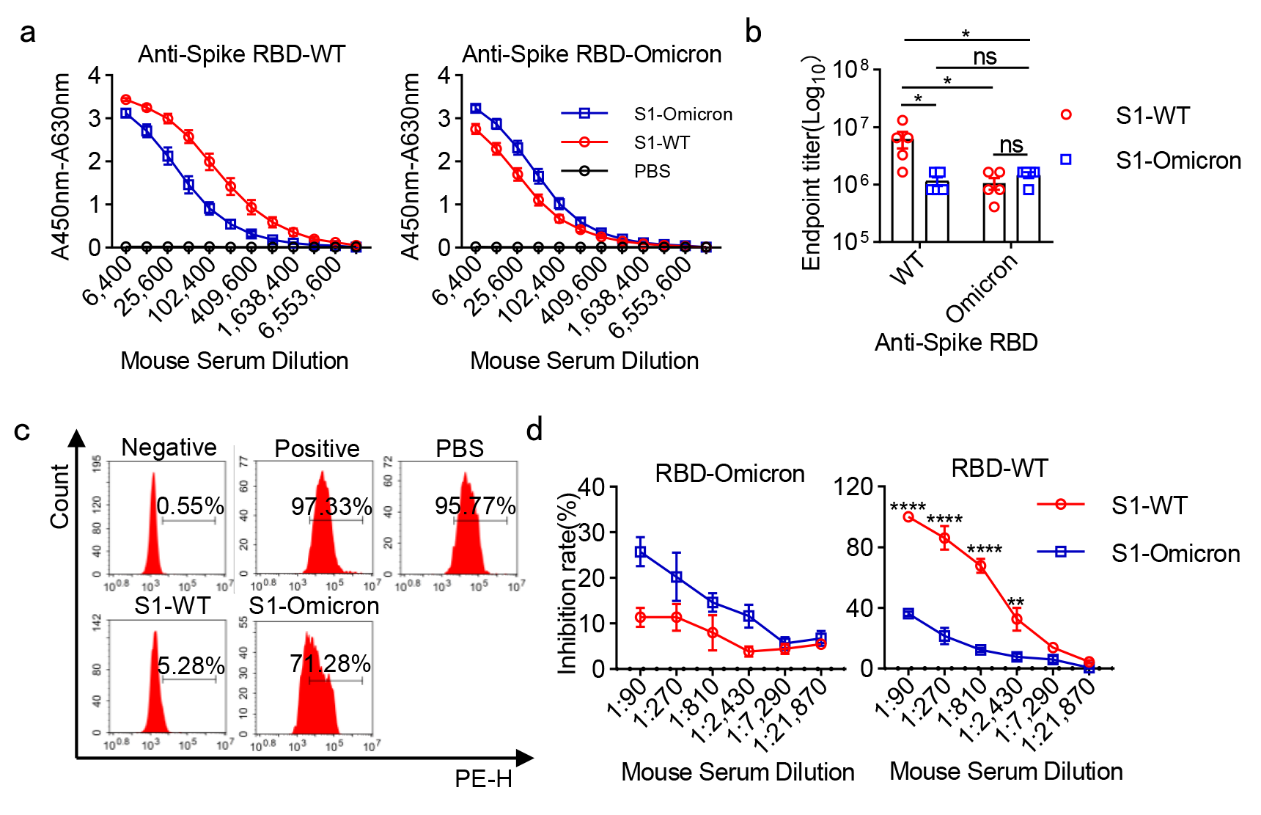
**
